# Supplementary material for: The Relationship between Subtypes of Health Literacy and Self-Care Behavior in Chronic Kidney Disease
Source: J Pers Med. 2021 May 22;11(6):447. doi: 10.3390/jpm11060447 (PMC8224639; doi:10.3390/jpm11060447)
Supplement: Supplementary file 1 [file jpm-11-00447-s001.zip › jpm-1143533-SI.pdf]

Supplement Table S1. The association of health literacy scores with self-care behavior in study subjects in univariate linear analysis

	Self-care behavior		Diet		Exercise	
	**β(95%CI)**	***p*-value**	**β(95%CI)**	***p*-value**	**β(95%CI)**	***p*-value**
Age (year)	0.05(-0.06,0.16)	0.41	-0.01(-0.05,0.03)	0.59	0.05(0.01,0.09)	0.03
Sex (female *v.s.* male)	-3.27(-6.09,-0.44)	0.02	-0.60(-1.63,0.43)	0.25	-1.36(-2.40,0.32)	0.01
Smoke (yes, %)	-9.10(-14.23,3.97)	0.001	-1.69(-3.59,0.21)	0.08	-1.20(-3.15,0.75)	0.23
Alcohol (yes, %)	-1.54(-8.11,5.04)	0.65	-1.18(-3.55,1.20)	0.33	0.15(-2.28,2.58)	0.90
Marital status (married, %)	-1.22(-4.69,2.25)	0.49	-0.42(-1.68,0.84)	0.51	0.36(-0.93,1.65)	0.58
Currently working (yes, %)	-2.60(-5.44,0.24)	0.07	-0.07(-1.11,0.96)	0.89	-1.24(-2.29,-0.19)	0.02
Education (senior high school or above, %)	2.65(-0.31,5.61)	0.08	1.82(0.76,2.87)	0.001	0.99(-0.11,2.09)	0.08
Hypertension (yes, %)	-1.13(-4.00,1.74)	0.44	-0.43(-1.47,0.61)	0.41	-0.51(-1.57,0.55)	0.34
Diabetes mellitus (yes, %)	-0.79(-3.73,2.16)	0.60	0.27(-0.80,1.34)	0.62	-0.63(-1.72,0.46)	0.25
Heart disease (yes, %)	-0.47(-4.11,3.17)	0.80	0.50(-0.82,1.81)	0.46	-0.69(-2.04,0.65)	0.31
Body mass index (kg/m^2)	-0.38(-0.74,-0.03)	0.04	-0.15(-0.28,-0.03)	0.02	-0.12(-0.26,0.01)	0.07
Number of health education sessions	0.21(0.09,0.34)	0.001	0.02(-0.03,0.07)	0.39	0.10(0.05,0.14)	<0.001
CKD duration (year)	0.51(0.16,0.85)	0.004	0.05(-0.08,0.17)	0.49	0.26(0.14,0.39)	<0.001
Health literacy (per score)	0.39(0.21,0.57)	<0.001	0.15(0.08,0.22)	<0.001	0.10(0.03,0.17)	0.006
eGFR (ml/min/1.73m^2)	0.01(-0.05,0.07)	0.71	-0.02(-0.04,0.01)	0.16	0.01(-0.01,0.03)	0.47
Hemoglobin (g/dl)	0.13(-0.56,0.81)	0.72	-0.12(-0.37,0.12)	0.32	0.24(-0.01,0.48)	0.06
Albumin (g/dl)	1.74(-1.52,5.01)	0.29	0.24(-0.94,1.42)	0.69	1.05(-0.15,2.25)	0.09
Uric acid (mg/dl)	0.93(0.171.69)	0.02	0.16(-0.14,0.45)	0.29	-0.05(-0.35,0.25)	0.75

Cholesterol (mg/dl)	-0.00(-0.04,0.03)	0.93	-0.00(-0.02,0.01)	0.71	-0.00(-0.02,0.01)	0.62
Log-formed triglyceride	-7.91(-13.95,-1.87)	0.01	-1.58(-3.81,0.65)	0.16	-2.56(-4.81,0.32)	0.03
Log-formed urine protein/creatinine ratio	-2.45(-4.79,-0.10)	0.04	-0.36(-1.21,0.49)	0.40	-1.16(-2.01,-0.31)	0.008
Log-formed glycated hemoglobin	-1.17(-2.40,0.06)	0.06	-0.47(-0.91,-0.03)	0.04	-0.43(-0.89,0.03)	0.07

Abbreviations: CKD, chronic kidney disease; eGFR, estimated glomerular filtration rate

Continue supplemental table S1

	**Home blood pressure monitoring**		**Smoking habits**		**Medication adherence**	
	**β(95%CI)**	***p*-value**	**β(95%CI)**	***p*-value**	**β(95%CI)**	***p*-value**
Age (year)	-0.04(-0.07,-0.01)	0.02	0.03(-0.00,0.05)	0.07	0.03(-0.03,0.08)	0.34
Sex (female *v.s.* male)	-0.27(-1.15,0.60)	0.54	--0.32(-0.41,1.06)	0.39	-1.35(-2.69,-0.02)	0.04
Smoke (yes, %)	-1.05(-2.67,0.56)	0.20	-	-	-0.89(-3.38,1.60)	0.48
Alcohol (yes, %)	0.06(-1.96,2.08)	0.96	-1.12(-2.81,0.57)	0.19	0.56(-2.55,3.66)	0.73
Marital status (married, %)	-0.40(-1.47,0.67)	0.46	-0.05(-0.94,0.85)	0.92	-0.72(-2.35,0.92)	0.39
Currently working (yes, %)	0.44(-0.44,1.32)	0.33	-0.98(-1.70,-0.25)	0.008	-0.75(-2.10,0.60)	0.28
Education (senior high school or above, %)	1.70(0.81,2.59)	<0.001	-0.43(-1.20,0.34)	0.27	-1.42(-2.82,-0.02)	0.04
Hypertension (yes, %)	0.07(-0.82,0.95)	0.88	0.24(-0.50,0.98)	0.52	-0.49(-1.85,0.86)	0.47
Diabetes mellitus (yes, %)	-0.80(-1.70,0.10)	0.08	0.25(-0.51,1.00)	0.53	0.13(-1.26,1.52)	0.85
Heart disease (yes, %)	-0.20(-1.32,0.92)	0.73	-0.31(-1.25,0.63)	0.52	0.23(-1.49,1.95)	0.79
Body mass index (kg/m^2)	-0.12(-0.23,-0.01)	0.04	-0.02(-0.11,0.08)	0.71	0.03(-0.14,0.20)	0.74
Number of health education sessions	0.01(-0.03,0.05)	0.75	0.03(0.00,0.07)	0.04	0.06(-0.00,0.12)	0.05

CKD duration (year)	0.03(-0.07,0.14)	0.54	0.07(-0.02,0.16)	0.12	0.10(-0.07,0.26)	0.25
Health literacy (per score)	0.07(0.01,0.13)	0.02	0.01(-0.04,0.06)	0.69	0.07(-0.03,0.15)	0.16
eGFR (ml/min/1.73m^2)	-0.01(-0.02,0.01)	0.48	0.01(-0.00,0.03)	0.08	0.01(-0.02,0.04)	0.43
Hemoglobin (g/dl)	0.02(-0.19,0.23)	0.85	-0.01(-0.19,0.16)	0.88	0.01(-0.31,0.33)	0.96
Albumin (g/dl)	-0.30(-1.30,0.71)	0.56	0.50(-0.33,1.34)	0.23	0.25(-1.30,1.79)	0.75
Uric acid (mg/dl)	0.15(-0.04,0.34)	0.12	0.05(-0.16,0.26)	0.63	0.55(0.18,0.93)	0.004
Cholesterol (mg/dl)	0.00(-0.01,0.01)	0.76	0.00(-0.01,0.01)	0.81	0.00(-0.02,0.02)	0.87
Log-formed triglyceride	-2.34(-4.23,-0.46)	0.02	-0.90(-2.45,0.66)	0.26	-0.53(-3.29,2.23)	0.71
Log-formed urine protein/creatinine ratio	-0.06(-0.26,0.15)	0.57	-0.52(-1.12,0.08)	0.09	-0.28(-1.40,0.85)	0.63
Log-formed glycated hemoglobin	-0.40(-0.80,0.00)	0.05	0.22(-0.08,0.52)	0.15	-0.10(-0.68,0.48)	0.74

Abbreviations: CKD, chronic kidney disease; eGFR, estimated glomerular filtration rate
